# Comparison of somatic variant detection algorithms using Ion Torrent targeted deep sequencing data

**DOI:** 10.1186/s12920-019-0636-y

**Published:** 2019-12-24

**Authors:** Qing Wang, Vassiliki Kotoula, Pei-Chen Hsu, Kyriaki Papadopoulou, Joshua W. K. Ho, George Fountzilas, Eleni Giannoulatou

**Affiliations:** 1 Victor Chang Cardiac Research Institute, Darlinghurst, Australia; 2 Department of Pathology, School of Health Sciences, Faculty of Medicine, Aristotle University of Thessaloniki, Thessaloniki, Greece; 3 Laboratory of Molecular Oncology, Hellenic Foundation for Cancer Research, Aristotle University of Thessaloniki, Thessaloniki, Greece; 4 School of Computer Science and Engineering, UNSW, Sydney, Australia; 5 School of Biomedical Sciences, Li Ka Shing Faculty of Medicine, The University of Hong Kong, Pokfulam, China; 6 St Vincent’s Clinical School, UNSW, Sydney, Australia; 7 Aristotle University of Thessaloniki, Thessaloniki, Greece

**Keywords:** Cancer genome, Ion torrent deep sequencing, Somatic variant calling, Methods evaluation, Read depth, Mutational signature

## Abstract

**Background:**

The application of next-generation sequencing in cancer has revealed the genomic landscape of many tumour types and is nowadays routinely used in research and clinical settings. Multiple algorithms have been developed to detect somatic variation from sequencing data using either paired tumour-blood or tumour-only samples. Most of these methods have been developed and evaluated for the identification of somatic variation using Illumina sequencing datasets of moderate coverage. However, a comprehensive evaluation of somatic variant detection algorithms on Ion Torrent targeted deep sequencing data has not been performed.

**Methods:**

We have applied three somatic detection algorithms, Torrent Variant Caller, MuTect2 and VarScan2, on a large cohort of ovarian cancer patients comprising of 208 paired tumour-blood samples and 253 tumour-only samples sequenced deeply on Ion Torrent Proton platform across 330 amplicons. Subsequently, the concordance and performance of the three somatic variant callers were assessed.

**Results:**

We have observed low concordance across the algorithms with only 0.5% of SNV and 0.02% of INDEL calls in common across all three methods. The intersection of all methods showed better performance when assessed using correlation with known mutational signatures, overlap with COSMIC variation and by examining the variant characteristics. The Torrent Variant Caller also performed well with the advantage of not eliminating a high number of variants that could lead to high type II error.

**Conclusions:**

Our results suggest that caution should be taken when applying state-of-the-art somatic variant algorithms to Ion Torrent targeted deep sequencing data. Better quality control procedures and strategies that combine results from multiple methods should ensure that higher accuracy is achieved. This is essential to ensure that results from bioinformatics pipelines using Ion Torrent deep sequencing can be robustly applied in cancer research and in the clinic.

## Background

Somatic cells in the tissues of our body carry the inherited diploid genotype, called the germline, with its variations described in recent large population genetics studies [1–3]. However, somatic cells also acquire genomic variants during life [4], which, in distinction to the germline, are called somatic variants. Regularly dividing progenitor cells for tissue renewal are particularly prone to the acquisition of somatic variants [5], i.e., single nucleotide variants (SNVs) and insertions/deletions (INDELs), as well as large structural variants and complex rearrangements. Accumulation of such variants in somatic cells, especially combinations with variants of deleterious potential for the function of key molecular pathways, may lead to the development of cancer [6]. The detection of tumour-specific variants is routinely performed in cancer research to investigate tumour biology and treatment options, and in the clinical setting to improve patient care in the context of personalised medicine. The most common strategy for the detection of somatic variation is through the application of next-generation sequencing (NGS) that allows an efficient, high-throughput characterisation of all types of genomic variation [7].

However, identifying somatic variants still remains a challenge due to their low allelic fraction as well as biological and technical issues such as contamination of the sample by non-malignant cells, tumour genetic heterogeneity and template degradation due to formalin-fixed and paraffin-embedded specimens (FFPE) [7, 8]. Among the various NGS platforms, Ion Torrent Targeted (IONT) sequencing is one of the commonly used technologies for targeted deep sequencing [9–11]. IONT sequencing typically reaches depth of more than 1000X even with FFPE templates, which makes it an ideal platform to identify somatic variants with variable allelic fraction. Yet, like all sequencing technologies, IONT has its drawbacks. It is PCR (primer) based for library construction and measures pH changes rather than optical signals, thus introducing biases and errors different from technologies that use probe-capture for library construction and fluorescence reads [12]. For instance, the quality of base calling accuracy generated by IONT platform is lower than other sequencing platforms, especially for INDEL events [13]. In addition, IONT is more vulnerable to homopolymer errors compared to other platforms [14].

Due to these features, to identify high-confidence somatic variants in IONT data, robust and accurate analytical methods need to be utilised. Multiple algorithms have been developed to detect somatic variation from sequencing data using either paired tumour-blood or tumour-only samples [15]. The performance of these methods has been extensively assessed in many benchmarking studies [16–22]. However, most somatic variant callers have been developed and often evaluated for the identification of somatic variation using Illumina sequencing datasets of moderate coverage. Although a previous effort has been made to evaluate some somatic variant callers on IONT data, the analysis only focused on SNVs and had a small sample size with only four cancer patients [23]. In our study, we applied three somatic mutation calling tools, Torrent Variant Caller (TVC), MuTect2 [24] and VarScan2 [25], on a large dataset derived from 208 paired blood – ovarian FFPE tumour and 253 ovarian FFPE tumour-only samples which were sequenced on an IONT platform with an AmpliSeq panel covering ~ 37 kb of the reference genome. We compared the variant calls returned by these methods for both SNVs and INDELs and investigated characteristics that affect the method concordance such as read depth and allelic fraction. Our study aims to provide recommendations for best practices for identifying somatic variation from IONT data.

## Results

We incorporated the three variants callers Torrent Variant Caller (TVC), MuTect2 and VarScan2 in a pipeline that normalises all resulting variants so that variants are left aligned and represented using the most parsimonious alleles [26], which allows comparison of both SNVs and INDELs. The details of the methods used are shown in Table 1. For tumour-only unpaired samples, only TVC and MuTect2 were compared as VarScan2 requires a paired tumour-normal input.

**Table 1:** Features of the variant calling tools that were compared for somatic variant detection from IONT data

Somatic variant caller	Statistical Method	Version	Variant Types	Somatic Variation Identification strategy	Input	Output
Torrent Variant Caller (TVC)	Subtraction	4.2	Somatic, germline	Call tumour and normal sample separately	Original BAM file	VCF file
MuTect2	Bayesian classifier	4.0	Somatic	Call paired tumour-normal or tumour-only samples	Sorted, indexed BAM file	VCF file
VarScan2	Fisher’s Exact Test	2.2.3	Somatic, germline	Call paired tumour-normal sample only	Pileup BAM file	VCF file

### Discrepancies of the three somatic variant callers

**Fig. 1:**
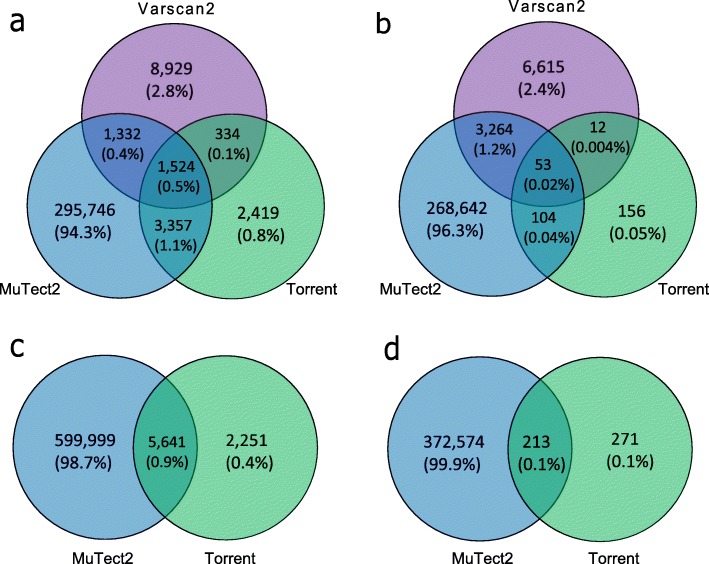
Concordance and discrepancy of the three somatic variant callers. The Venn diagrams illustrate the total counts and comparison of somatic mutations called by each of the somatic variant callers for **a** SNVs and **b** INDELs identified from paired tumour-blood samples as well as **c** SNVs and **d** INDELs from unpaired tumour-only samples. The numbers in the parenthesis reveal the percentage of variants against all the variants

We observed low concordance among the three somatic callers, TVC, MuTect2 and VarScan2 for both SNVs and INDELS (Fig. 1a and b). In total, 313,641 SNVs were identified by the three variant calling methods in ovarian tumours and matched blood paired samples from 208 patients. There was large variation in the number of SNV calls returned by the three callers (Fig. 1a). In brief, 301,959 SNVs were detected by MuTect2, while VarScan2 called 12,119 SNVs and TVC called 7634 SNVs across all samples. MuTect2 had the largest number of unique variants (295,746, 94.3%) that did not overlap with other methods, whereas VarScan2 had 8929 unique variants (2.8%) and TVC called 2419 unique variants (0.8%). The common variants for all three methods were 1524, only 0.5% of the total SNVs. Though TVC had the lowest number of variant calls, it had the largest percentage of calls that agreed to other methods (20%).

For INDELs, fewer variants (278,846) were called by all the three algorithms in total (Fig. 1b). Similar to SNVs, MuTect2 had the highest number of both unique calls (268,642) and overall calls (272,063). Moreover, 16% of TVC calls were common calls whereas merely 0.02% of MuTect2 calls and 0.5% of VarScan2 calls were shared by the other two methods. In comparison to SNV calling, lower concordance across different algorithms was revealed with roughly 0.02% of all the variants being common across all three methods.

For the unpaired tumour-only samples, only TVC and MuTect2 algorithms could be applied as VarScan2 requires a matched normal (germline) sample for somatic variant calling. Variants previously considered as somatic may in fact be present at low frequency in the human population [27]. The 1000 Genomes Project shows that more than 95% of an individual’s germline mutations variants including SNVs and INDELs occur at a frequency of more than 0.5% in the general population [28]. Such variants are considered common variants and are mostly not associated with cancer development. To identify potential somatic variants in this series, we selected all variants that are rare in control population databases (Minor Allele Frequency (MAF) < 0.1% across 1000Genomes, ExAC and gnomAD) [1–3] after functional annotation with ANNOVAR [29]. Overall, 607,891 SNV variants and 373,058 INDEL variants were discovered. Likewise, MuTect2 still had the largest amount of SNV calls, more than 98% of which were not returned by TVC (Fig. 1c). For SNV and INDEL calling, the common variants were 71 and 44% of TVC calls, respectively. In comparison with SNV calling, lower agreement was revealed in INDEL detection (Fig. 1d). Specifically, approximately 0.1% of the INDEL candidates were common calls whereas 0.9% of the SNV calls were common calls between these two algorithms.

### Distribution of variant allele frequency (VAF) and read depth of SNVs

**Fig. 2:**
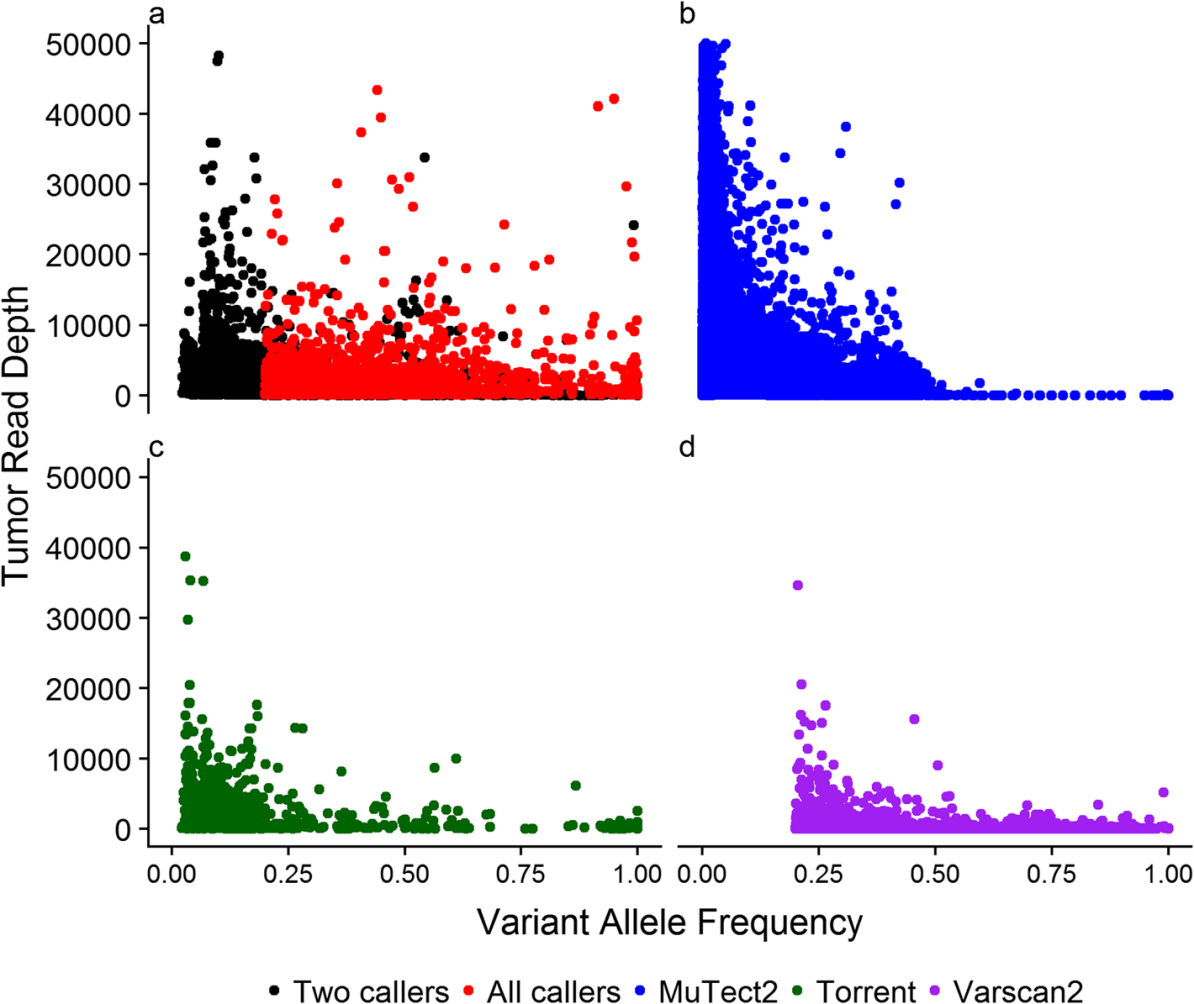
Comparison between SNVs identified by the three somatic variant callers. The Y-axis of each scatter plot indicates the read depth of SNVs in tumour tissue sample, the X-axis shows the VAF of each SNV. The dots represent SNVs from tumour-blood pairs. The colour indicates the variant calling method. **a** All callers: SNVs identified by all three callers; Two callers: SNVs identified by any two of the three callers. **b** MuTect2: SNVs uniquely identified by MuTect2. **c** Torrent: SNVs uniquely identified by TVC. **d** VarScan2: SNVs uniquely identified by VarScan2

To further compare the performance of the variant callers, we investigated the relationship between variant allele frequency (VAF) and read depth for each set of algorithm-specific SNVs as well as the common SNVs across two or all three callers in the 208 paired tumour-blood samples (Fig. 2). The unique variants identified by each method show distinct VAF distribution. MuTect2 identified variants with lower VAF (mean = 0.019, sd = 0.04) compared to TVC (mean = 0.16, sd = 0.18) and VarScan2 (mean = 0.42, sd = 0.21). The latter by default does not identify any somatic variation with VAF < 0.2. The somatic variation detected by TVC showed a higher uniformity across VAF levels. We observed a high inverse correlation between the read depth and VAF in somatic variants called by MuTect2 only (Spearman rho = − 0.53, *p*-value < 2.2 × 10^− 16^), moderate inverse correlation for somatic variants called by IONT only (Spearman rho = − 0.34, *p*-value < 2.2 × 10^− 16^), while the somatic variants identified by VarScan2 only did not exhibit such correlation (Spearman rho = − 0.02, *p*-value =0.053).

The majority of the variants called only by MuTect2 had low VAF (98% of variants had VAF < 0.1). Conversely, the set of calls detected by TVC had widespread allele frequency at lower sequencing depth. This depicts that TVC performed well in a variable range of VAF and did not overcall in regions of high read depth. Though of similar read depth to TVC, VarScan2 missed mutations that have allele frequency from 0 to 0.2 due to the default settings of the algorithm. In contrast to the other two callers, VarScan2 identified more variants with higher allele fraction (27.0% of the variants with VAF > 0.5 in contrast to IONT with 6.1% and MuTect2 with 0.08%). In terms of the mutations called by any two methods and called by all methods, large overlap existed for variable VAF and read depth. All callers identified variants at a wide VAF range (0.02 to 1.00) and only a small number of variants in high-depth regions (319 SNVs with read depth higher than 4000).

### Mutational signature of variants

**Fig. 3:**
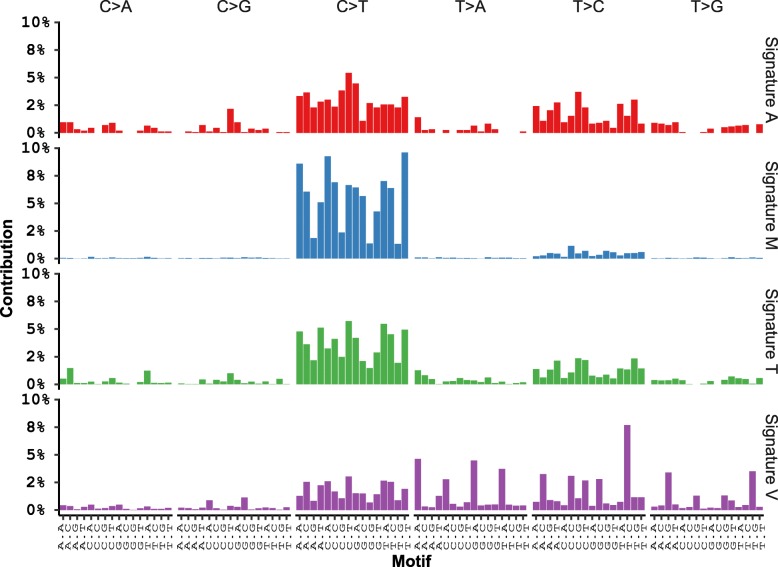
Mutational signatures of SNVs called from paired samples. The Y-axis reveals the frequency of each mutation type in the whole human genome, the X-axis indicates the mutation types according to 96 possible substitution types defined by the bases 5′ and 3′ to the mutated base. Signature A: mutational signature inferred from SNVs detected by all three callers; Signature M: mutational signature inferred from all the SNVs detected by MuTect2; Signature T: mutational signature inferred from all the SNVs detected by TVC; Signature V: mutational signature inferred from all the SNVs detected by VarScan2

To further evaluate the performance of each somatic variant calling tool, mutational signature analysis was performed. The patterns of somatic variants called by different variant callers were described by mutational signatures according to 96 possible substitution types defined by the bases 5′ and 3′ to the mutated base [30]. Four novel mutational signatures were inferred from caller-unique variants as well as common variants identified in 208 paired tumour-blood ovarian samples (Fig. 3). The somatic signature of MuTect2 calls (Signature M) was dominated by C > T substitutions. TVC calls and common calls also featured with high proportions of C > T substitutions in their mutational signatures (Signature T and Signature A). However, unlike MuTect2, they also exhibited some lower representation of other substitution types (such as T > C, C > A and T > G). Conversely, the mutational signature of the variants called only by VarScan2 (Signature V) was enriched for T > A and T > C substitutions and peaked at T > C at TNC trinucleotides.

We examined the correlations between each mutational signature predicted in our study and the ones reported for ovarian cancer from the Catalogue Of Somatic Mutations In Cancer (COSMIC) (Signature 1, Signature 3, Signature 5) [31]. COSMIC is the world’s largest and most comprehensive resource for exploring the impact of somatic variants across a broad range of human cancers [31]. Signature 1 has been found in all cancer types and in most cancer samples and is the result of an endogenous mutational process initiated by spontaneous deamination of 5-methylcytosine. Signature 3 has been found in breast, ovarian, and pancreatic cancers and is associated with failure of DNA double-strand break-repair by homologous recombination. Signature 5 has been found in all cancer types and most cancer samples, but it is aetiology is unknown.

The correlation between our predicted mutational signature patterns and the reference Signature 1 was found to be the highest for Signature A (Table 2, *r* = 0.44, *p*-value< 0.05), followed by Signature T (Table 2, *r* = 0.34, *p*-value< 0.05). Signature V was least correlated with Signature 1 (Table 2, *r* = 0.04, *p*-value< 0.05). A similar pattern was also shown in the correlation between Signature 5 and the four mutational signatures (Table 2). Unlike Signature 1 and Signature 5, Signature 3 exhibited weak and nonsignificant correlation with the four inferred mutational signatures.

**Table 2:** Correlation between detected mutational signatures and the COSMIC ovarian cancer mutational signatures

	Signature 1 Spearman rho (*p*-value)	Signature 3 Spearman rho (*p*-value)	Signature 5 Spearman rho (*p*-value)
Signature A (All)	0.44 (< 0.05)	0.06 (0.59)	0.64 (< 0.05)
Signature M (MuTect2)	0.27 (< 0.05)	0.09 (0.37)	0.46 (< 0.05)
Signature T (TVC)	0.34 (< 0.05)	0.08 (0.43)	0.57 (< 0.05)
Signature V (VarScan2)	0.04 (< 0.05)	−0.01 (0.90)	0.24 (< 0.05)

### Comparison with COSMIC somatic variation

We investigated the overlap of all SNVs identified in the paired tumour-blood samples with all coding and non-coding variation reported in COSMIC v87. In this analysis, the variants present in COSMIC are regarded as reference for the estimation of sensitivity of the three variant callers. We found that 3620 of 28,246 (12.8%) unique somatic variants detected using MuTect2 were also reported as somatic variants in the COSMIC database, whereas 64 of 732 (8.7%) IONT variants and 95 of 3044 (3.1%) VarScan2 variants were found in the COSMIC database. SNVs detected by all variant callers showed the highest level of overlap with COSMIC database (156 of 592, 26.4%).

### Concordance of pathogenic variants in frequently mutated genes

Finally, we compared the number of potentially pathogenic variants identified by each variant caller in five genes that are frequently mutated in cancer, particularly in ovarian cancer: *BRCA1*, *BRCA2*, *KRAS*, *PIK3CA* and *TP53* [32, 33]. Variants were considered pathogenic if they met the following criteria: (1) their minor allele frequency (MAF) was below 0.1% across all three large control databases 1000 Genomes, ExAC and gnomAD [1, 2]; (2) the variants were either nonsynonymous, stopgain or stoploss or frameshift deletions or insertions and (3) the variants were reported as pathogenic in ClinVar [34] or predicted to be deleterious by FATHMM [35] or have occurred in either breast or ovarian cancer samples according to COSMIC v88 [31].

The number of pathogenic variants detected by each variant caller as well as their intersection are shown in Tables 3 and 4 for paired and unpaired samples respectively. For both categories, MuTect2 identified the highest number of pathogenic mutations in all the five genes and in a larger number of samples compared to the other callers. However, the number of common variants identified by all callers was substantially different to the number of variants identified by MuTect2, which again supported that the latter might be more prone to false positive calls. In paired samples, though TVC identified fewer overall calls compared to VarScan2, it called more unique pathogenic mutations in most of the five genes except *BRCA2.* In unpaired samples, the number of pathogenic variants identified by TVC was similar to those identified by both TVC and MuTect2. All algorithms called more variants in *BRCA1* and *TP53* in tumours than in *BRCA2*, *KRAS* and *PIK3CA*, in line with the reported incidence for such mutations in ovarian cancer.

**Table 3:** The number of pathogenic mutations identified in 208 paired tumour-blood samples in five genes that are frequently mutated in ovarian cancer. The number of unique mutations is shown and in brackets the number of samples with at least one mutation is shown

	*BRCA1*	*BRCA2*	*KRAS*	*PIK3CA*	*TP53*
MuTect2	1056 (*n* = 207)	364 (*n* = 207)	51 (*n* = 157)	45 (*n* = 137)	738 (*n* = 208)
TVC	126 (*n* = 83)	30 (*n* = 38)	10 (*n* = 31)	16 (*n* = 27)	139 (*n* = 139)
VarScan2	94 (*n* = 188)	57 (*n* = 194)	7 (*n* = 19)	10 (20)	111 (*n* = 161)
Common	33 (*n* = 77)	8 (*n* = 34)	7 (*n* = 17)	7 (*n* = 15)	81 (*n* = 122)
Common (%)	2.9% (37%)	2.1% (16.4%)	13.7% (10.8%)	14.9% (10.6%)	10.7% (57.8%)

**Table 4:** The number of pathogenic mutations identified in 253 unpaired tumour samples in five genes that are frequently mutated in ovarian cancer. The number of unique mutations is shown and in brackets the number of samples with at least one mutation is shown

	*BRCA1*	*BRCA2*	*KRAS*	*PIK3CA*	*TP53*
MuTect2	1190 (*n* = 243)	390 (*n* = 248)	56 (*n* = 202)	47 (*n* = 186)	820 (*n* = 246)
TVC	164 (*n* = 89)	54 (*n* = 42)	11 (*n* = 34)	18 (*n* = 27)	215 (*n* = 175)
Common	163 (*n* = 85)	52 (*n* = 42)	11 (*n* = 34)	14 (*n* = 24)	210 (*n* = 173)
Common (%)	13.7% (34.4%)	13.3% (16.9%)	19.6% (16.8%)	27.5% (12.7%)	25.5% (69.8%)

## Discussion

Somatic variant detection is a fundamental step and thus extremely crucial in most cancer sequencing projects. The main challenge falls in identifying potential false positive and false negative calls. In our study, we utilized a large IONT sequencing dataset of ovarian tumours to comprehensively evaluate three somatic mutation callers. We found that different callers considerably varied on variant detection and that aggregating results from various tools offered improvement in the variant calling performance.

Our study reported low concordance across the methods for somatic SNVs calling, which has also been highlighted in several previous studies for other types of NGS datasets [19, 20, 36]. Among the three callers, MuTect2 identified the largest number of somatic SNVs, but they were in poor agreement with other tools. This suggests that MuTect2 is less stringent and most of the calls are likely to be false positives for IONT data. MuTect2 utilises the somatic genotyping engine of the original MuTect algorithm which was developed to have higher sensitivity for mutations with allelic fractions as low as 0.1 and below [24]. However, it is questionable whether such sensitivity is practically needed when interrogating diagnostic and actionable variants in tumour tissues [37]. For example, in a tissue sample with malignant cell DNA content of 50%, the rest comes from non-malignant cells. Detecting a pathogenic or actionable variant at an allelic fraction of 0.1 or even 1, does not necessarily determine whether it is present in malignant or non-malignant cells. In comparison to MuTect2, TVC returned the lowest number of SNV calls, most of which overlapped with calls from other tools. This confirmed the observation reported in a previous study which suggested the reasonable trade-off between sensitivity and specificity of TVC [23]. One advantage of our study is that we also investigate the discrepancies in INDEL calling. MuTect2 also showed the least concordance among methods in detecting INDELs. Additionally, lower agreement among all three tools was also revealed, which indicated a greater challenge to call INDELs compared to SNVs. Another strength of our study is that we also compared the performance of callers in unpaired tumour-only samples, as most of the clinical samples submitted for NGS are tumour-only FFPE samples [38]. We observed similar performance of MuTect2 and TVC in detecting both SNVs and INDELs in unpaired samples. For unpaired samples, the inference on whether a mutation is somatic was based on their frequency in the population. Although this was an approximation, this gave us a way to compare the two methods in their ability to call somatic variants. The discrepancies we observed among all somatic variant callers might be due to the different statistical models and assumptions of these methods. More importantly, both MuTect2 and VarScan2 were developed and optimised for Illumina NGS datasets rather than IONT deep sequencing data that exhibit much higher coverage and a different error structure.

Merely comparing the number of variant calls is not sufficient and previous studies have indicated that discrepancies across various variant callers are largely due to the variant characteristics identified in the tumour samples [21]. Therefore, we also focused on the properties (VAF and read depth) of both common and unique variants across different methods in paired tumour-normal samples. By investigating the relationship between VAF and read depth of somatic SNVs, it was revealed that the algorithms show distinct distributions. MuTect2 favoured calling variants at low depth and low VAF as it has also been reported previously [21]. Due to the deep read depth of IONT data, MuTect2 also returned a large number of mutations with high coverage. Hence, MuTect2 is relatively more sensitive to coverage change and the number of calls predicted is dependent on the read depth. Although higher sequencing depth allows detection of variants with lower VAF, the strong correlation observed in calls only by MuTect2 indicates that these are potentially false positive calls and the algorithm is very sensitive to base mismatches present in the mapped reads. Assuming adequate coverage to identify all true somatic variation within a sample, such a correlation is not expected. Both TVC and VarScan2 called variants with relatively lower tumour read depth compared with MuTect2. Nevertheless, VarScan2 showed advantage in identifying somatic SNVs with relatively high allelic frequency, which is also confirmed in other studies [18, 21]. In terms of variant allele frequency, VarScan2 was unable to detect variants with allelic fraction lower than 0.2, based on its default design, and thus any mutations below this threshold were suppressed [25]. It has also been reported that VarScan2 outperformed other variant calling tools when allele frequency is higher than 0.35 and MuTect2 performs better for variants with allele frequency lower than 0.35 [16]. As a result, this makes VarScan2 a good method to complement the other two callers. Finally, by investigating the relationship between VAF and read depth it was also revealed that the common variants across all methods exhibit a wide range of VAF as well as relatively high coverage. This suggests that by combining the results of all three callers we can exploit the advantages of each caller and thus possibly provide a more reliable output.

One limitation of our study is the lack of experimental validation datasets and ground truth. As a proxy, we applied mutational signature analysis on unique and common variants from each method to explore the reliability of somatic variant calling by correlating with known signatures of ovarian cancer. Somatic mutations reflect mutagenic processes caused by carcinogenic agents which accumulate during the development of cancer [30]. By far, up to 30 mutational signatures have been derived from public dataset across 40 different types of human cancers to characterize these mutagenic events [31]. As only validated mutational signatures were included, the publicly available somatic mutation signatures were regarded as “gold standard” and were employed to compare the performance of different callers.

The signature plots provided a brief overview of the performance of each variant calling tool on identifying variants. The prominence of C > T substitutions in MuTect2 calls suggested that MuTect2 might have distinctive specificity of certain mutation types and fail to detect other substitution types from IONT data. The mutational signatures from TVC and common calls had similar features. As also illustrated from our previous concordance comparison, TVC had the highest agreement with other tools. VarScan2 showed a unique pattern of substitution, which also confirmed its low concordance with other callers from previous results in our study. We should note that the signature observed in our samples could be influenced by the fact that our samples are FFPE which lowers DNA quality and increases deamination mutation signature [39].

In COSMIC, ovarian cancer is characterized by Signature 1, Signature 3 and Signature 5. Signature 1 and Signature 5 have been observed in 25 cancer types and feature age-related mutagenesis, whereas Signature 3 is more cancer-type specific and has been identified in breast, ovarian and pancreatic cancer [30]. The strongest positive correlation displayed between Signature A and both Signature 1 and Signature 5 agreed with our results as well as previous research showing that the combination of three callers perhaps captures the most accurate variants [21, 40]. The second highest correlation with Signature 1 and Signature 5 was revealed in Signature T from TVC calls. This indicated that TVC is more likely to produce reliable results than the other callers. VarScan2 calls exhibited the lowest correlation with any of the ovarian cancer signatures, possibly due to its failure to call variants with VAF lower than 0.2. Thus, it missed the somatic variants which have low allele frequency. Signature 3 is not found to be significantly correlated with the signatures we modelled, which might be caused by the different ovarian cancer types in our cohort.

In addition, we also compared the SNVs detected by different variant callers with the variation in COSMIC v87. For this analysis, the variants present in this database are regarded as reference for the estimation of sensitivity of the three variant callers as somatic variation can be recurrent across samples. Similarly to other results, variants called by all three callers represented the highest agreement with COSMIC. This offers additional evidence that the consensus of variant calling algorithms provides more true positive calls.

Finally, we compared the number of pathogenic variants in five genes that are frequently mutated in ovarian cancer *BRCA1*, *BRCA2*, *KRAS*, *PIK3CA* and *TP53*. This analysis highlighted that care should be taken in identifying pathogenic mutations using the state-of-the-art variant callers as their concordance in the number and type of mutations identified per gene and per tumour was very low. Strict quality control procedures should be applied to eliminate false positives given that mutations in these genes can drive treatment decisions in the clinic.

A further limitation of our study is that in order to obtain comparable outputs as well as satisfying the developer’s original purpose, the default settings of the variant calling tools were accepted. The only modifications in the default parameters of MuTect2 and VarScan2 were made to allow input samples of high coverage, which is characteristic of IONT data. However, if the features of the dataset and the specific goal of the project are available, further tuning the parameters should not be neglected to improve the reliability of the results [15]. Thus, additional caller tuning and filtering is crucial to control false positive results and optimize somatic mutation discovery for future cancer research. In addition, automation does not cover the final variant evaluation for clinical purposes, even with validated panels.

The advantage of our study is that several approaches were utilised to comprehensively evaluate the somatic variant callers. Nonetheless, rather than simulated data, we used real datasets, which illustrates the true biological characteristics of the sample and sequencing errors and illustrates the performance of the variant callers in a real setting. Moreover, our sample size is larger than most existing variant calling tool evaluation studies with 208 paired and 253 unpaired samples [23].

## Conclusion

In conclusion, our study first characterized the discrepancies among popular somatic variant callers in IONT data in a large ovarian cancer cohort. The consensus of different somatic variant callers was suggested to have the most reliable output. TVC showed the highest concordance with the other methods indicating a good performance. Our study points out the insights of combined strategies to achieve higher sensitivity, which can be crucial in future cancer research and in personalized medicine. It also highlights that caution should be taken when applying methods developed for analysing data produced by a specific sequencing technology.

## Methods

### Sequencing

We analysed targeted NGS genotype data from 461 FFPE tumor tissues and 208 peripheral blood sample that had been retrieved from the biorepository by the Hellenic Cooperative Oncology Group (HeCOG). Sample processing was accomplished at the Laboratory of Molecular Oncology (MOL by Hellenic Foundation for Cancer Research / Aristotle University of Thessaloniki, Greece). Tissues were histologically reviewed and further used for the construction of tissue microarrays (TMA) that included 3 X 1.5 mm diameter cores per tumour. DNA was extracted with the QIAamp® DNA Mini kit (Qiagen, Hilden, Germany) from 8um TMA core sections. Tumor cell DNA content was ≥50% in 75% of the samples. For sequencing, we used a previously described custom Ampliseq panel (IAD75668_167; Applied Biosystems / Thermo Fisher Scientific, Paisley, UK) targeting a total area of 36.8Kb and 330 amplicons including coding regions in 40 genes [33]. Twenty ng FFPE DNA were used for library construction and sequencing was accomplished with the Ion Torrent Proton sequencing platform (Thermo Fisher).

### Somatic variant calling

The binary alignment map (BAM) files were generated by the Ion Torrent Proton sequencing platform (Thermo Fisher) and aligned by Ion Torrent Variant Server (ITV) with Torrent Suite v5.0.2. The mean read depth was 4250.8 across all samples and amplicons. Three somatic variant callers Torrent Variant caller (TVC) 4.2, MuTect2 4.0 and VarScan2 2.2.3 were applied on tumour and matched blood samples. For the unmatched samples, only Torrent Variant caller and MuTect2 were used.

TVC is a genetic variant caller supported by ITV and deals with the BAM file from the Ion Torrent sequencing platforms directly. Variants were identified by subtracting normal sample calls from tumour sample calls. Then the tumour and blood VCF output files were merged by VCFtools [41] and somatic variants were selected by finding mismatches between the genotypes of tumour and normal samples.

MuTect2 is a somatic SNP and indel caller that combines the somatic genotyping engine of the original MuTect [24] with the assembly-based machinery of HaplotypeCaller provided by GATK [42, 43]. It detects only somatic mutations in NGS data using a Bayesian classifier approach. The input of MuTect2 requires BAM files sorted and indexed by SAMtools (v 1.3.1) [44]. After removing low quality sequenced data, the variants were detected by comparing the likelihood of the site to be variant to sequencing noise and filtered with a panel of normal samples to reduce miscalled germline calls. Finally, a classification of somatic calls is performed using similar classifier as variant detection but with more stringent threshold [24]. GATK (v3.7) MuTect2 was run with default parameters. Due to the deep read depth of IONT data, the *-max-reads-per-alignment-start* parameter was set to 0, which disabled down-sampling and took all the reads into account. The output is VCF files with only somatic variants.

VarScan2 reads SAMtools mpileup output from the normal and tumour paired samples simultaneously and heuristically calls a genotype at every position achieving the coverage and quality thresholds [25]. Modification was made with *mpileup* command to adjust for the deep depth of IONT data: 1) the parameter *–d* (the maximum number of reads to be read at one position in one BAM file) was set to 50,000; 2) the parameter *–L* (the threshold of the average per-sample depth to skip INDEL calling) was set to 1000. If a variant is identified in either normal or tumour sample, Fisher’s exact test was then used to classify the variant into somatic (enriched in tumour), germline (enriched in both tumour and normal), or loss of heterogeneity (LOH) (enriched in normal). The SNVs and INDELs were called separately by VarScan2 and then concatenated using VCFTools [41]. Since VarScan2 calls both somatic and germline variants, the somatic variants were further selected by those labelled as “SOMATIC” in the INFO field in the final VCF files.

For tumour-only unpaired samples, only TVC and MuTect2 were applied with same settings as paired samples. To identify somatic mutation in the unpaired tumour-only samples, we selected all variants that are rare in control population databases (Minor Allele Frequency (MAF) < 0.1% across 1000Genomes, ExAC and gnomAD) [1, 2] after functional annotation with ANNOVAR [29].

### Methods concordance and performance assessment

Before the comparison of somatic variant calling algorithms, the VCF files were normalized by Variant Tools (Vt) to ensure the variants are parsimonious, left aligned as well as reordered [26]. In addition, Vt was also used to decompose multi-allelic variants into bi-allelic or its constituent SNPs to allow further allelic comparison between different call sets.

Subsequently, to evaluate the concordance of the variant callers, Venn diagrams were generated for somatic SNVs and INDELs respectively in both 208 paired samples and 253 unpaired tumour-only samples by Venny (v 2.1.0) [45]. To further characterize the concordant and discrepant calls from different algorithms, scatter plots were generated with variant allele frequency (VAF) and read depth of each somatic SNV called by only one caller, any two callers or all the callers.

Finally, mutational signature analysis was also performed to evaluate the variant calling tools. The patterns of somatic variants called by different variant callers were described by mutational signatures according to 96 possible substitution types defined by the bases 5′ and 3′ to the mutated base [30]. The mutational signature was inferred in nonnegative matrix factorization approach using SomaticSignature R package across all 208 paired samples [30, 46]. Moreover, the Spearman’s correlation was calculated to compare the identified signature patterns in our study with the ovarian cancer mutational signatures currently reported in the Catalogue Of Somatic Mutations In Cancer (COSMIC) [31]. The variations present in COSMIC were also compared with the SNVs detected in all paired tumour-blood samples to estimate the true positive calls.

## Data Availability

The datasets used and/or analysed during the current study are available from the corresponding author on reasonable request.

## Abbreviations

COSMIC: Catalogue Of Somatic Mutations In Cancer
FFPE: Formalin-fixed and paraffin-embedded specimens
INDELs: Insertions/deletions
IONT: Ion Torrent Targeted
MAF: Minor Allele Frequency
NGS: Next-generation sequencing
SNVs: Single nucleotide variants
TVC: Torrent Variant Caller
VAF: Variant allele frequency
